# Effect of Combined Treatment of Ketorolac and Remote Ischemic Preconditioning on Renal Ischemia-Reperfusion Injury in Patients Undergoing Partial Nephrectomy: Pilot Study

**DOI:** 10.3390/jcm7120470

**Published:** 2018-11-23

**Authors:** Hae Keum Kil, Ji Young Kim, Young Deuk Choi, Hye Sun Lee, Tae Kwang Kim, Ji Eun Kim

**Affiliations:** 1 Department of Anesthesiology and Pain Medicine, Anesthesia and Pain Research Institute, Yonsei University College of Medicine, Seoul 03722, Korea; hkkil@yuhs.ac (H.K.K.); KIMJY@yuhs.ac (J.Y.K.); 2 Department of Urology, Yonsei University College of Medicine, Seoul 03722, Korea; YOUNGD74@yuhs.ac; 3 Biostatistics Collaboration Unit, Yonsei University College of Medicine, Seoul 03722, Korea; HSLEE1@yuhs.ac; 4 Department of Anesthesiology and Pain Medicine, Ajou University School of Medicine, Suwon 164, Korea; itsagoodtimee@gmail.com

**Keywords:** acute kidney injury, ischemic preconditioning, ketorolac

## Abstract

We evaluated postoperative renal function in patients with/without combined therapy of ketorolac and remote ischemic preconditioning during partial nephrectomy. Sixteen patients were randomly allocated to either the ketorolac combined with RIPC group (KI, *n* = 8) or control group (*n* = 8). The KI group received both remote ischemic preconditioning before surgery and intravenous ketorolac of 1 mg/kg before renal artery clamping. Renal parameters were measured before induction, after anesthesia induction, and 2, 12, 24, 48, and 72 h after renal artery declamping. Acute kidney injury was assessed by Acute Kidney Injury Network criteria. The estimated glomerular filtration rate decreased in both groups, but then increased significantly at 48 h and 72 h after declamping only in the KI group compared to 24 h (*p* = 0.001 and *p* = 0.016). Additionally, it was higher at 48 h and 72 h after declamping in the KI group compared to the control group (*p* = 0.025 and *p* = 0.044). The incidence of acute kidney injury was significantly reduced in the KI group (13%) compared to the control group (83%) (*p* = 0.026). FE_Na_ was markedly increased at 2 h after declamping, and recovered in both groups, but it was more significant at 12 h after declamping in the KI group (*p* = 0.022). Urinary N-acetyl-1-β-D-glucosoaminidase and serum neutrophil gelatinase-associated lipocalin were similar (*p* = 0.291 and *p* = 0.818). There is a possibility that combined therapy of ketorolac and remote ischemic preconditioning prior to ischemia may alleviate renal dysfunction and reduce the incidence of acute kidney injury in patients undergoing partial nephrectomy.

## 1. Introduction

Partial nephrectomy (PN) is a standard procedure for a small kidney tumor. Although the benefit of PN is the better preservation of renal function compared to the radical nephrectomy, a part of viable parenchyma is removed, surrounding tissue is damaged by cautery or compression, and the remaining parenchyma usually experiences the ischemia [1]. Chronic kidney disease (CKD) was developed in one-third of patients after PN, who had a normal preoperative glomerular filtration rate (GFR) [2]. Renal vascular clamping, which is frequently performed to secure bloodless surgical fields during PN, renders the kidney susceptible to tissue ischemic injury. Ischemia-reperfusion initiates a cascade of events of tissue injury and death [1,3]. Because ischemia is the main risk factor for the impairment of preserved renal tissue, various interventions have been conducted for prevention of ischemia-related postoperative renal dysfunction [4,5]. 

Among several mechanisms in progression to acute kidney injury, oxidative stress plays an important role by activation of inflammation via proinflammatory cytokine release and inflammatory cell accumulation in tissues [6]. There are four response phases to ischemia-reperfusion injury on the kidney (adaptation, extension, maintenance, and repair phases). During an extension phase with the restoration of renal blood flow after ischemia, numerous inflammations are triggered. Then, inflammation-induced cell death is characterized during the maintenance phase despite the resolution of hypoxia [7]. 

Non-steroidal anti-inflammatory drugs (NSAID) may have some role in inflammatory events through an inhibition of abnormal cyclooxygenase (COX) activity, reactive oxygen species (ROS) generation, and prostaglandin (PG) production [7,8,9]. On the other hand, several studies have shown that remote ischemic preconditioning (RIPC), which is defined as transient brief episodes of ischemia at a remote site before a subsequent ischemia-reperfusion injury of the target organ, reduced the organ damage in several pathways; signal transduction pathways, neuronal and humoral pathways, and anti-inflammatory pathways [10,11].

The aim of this pilot study is to evaluate the postoperative renal function in patients with and without combined therapy of NSAID (ketorolac) and RIPC during open PN.

## 2. Materials and Methods

This randomized, single blinded, prospective and parallel-group controlled pilot study was conducted on patients undergoing elective open PN. The present study was approved by the Institutional Review Board (4-2012-0485) and registered at ClinicalTrials.gov (NCT 01836406). Written informed consent was obtained from all participants.

In all, 16 patients with a single renal mass of 4–7 cm of size were enrolled. Criteria for exclusion were solitary kidney, age ≥ 70 years, liver or renal dysfunctions, history of renal surgery, multifocal tumors, coagulopathy, hypersensitivity to NSAID, peptic ulcer, chronic alcoholism, peripheral neuropathy, diabetes, or hypertension medicated with angiotensin receptor blocker or diuretics.

Patients were randomly allocated to either the ketorolac combined with RIPC group (KI, *n* = 8), or control group (*n* = 8) by randomization. In the KI group, RIPC was implemented after anesthetic induction and completed before the start of surgery by using a tourniquet (Zimmer ATS 2000; Zimmer, Dover, OH, USA) applied to the upper arm of dependent side. Three cycles of cuff inflation (200 mmHg, 5 min) and deflation (10 min) were done. During surgery, 1 mg/kg of intravenous (IV) ketorolac tromethamine (Keromin^®^, CJ Pharmaceutical Co., Ltd., Seoul, Korea) was given before 30 min of renal artery clamping. In the control group, a tourniquet was placed without inflation or deflation throughout the procedure, and an equal volume of normal saline as a placebo was given in same manner. An independent investigator prepared the study’s drug solutions and provided the coded drug to the anesthetic provider before anesthesia. A tourniquet was manipulated by a primary investigator. The anesthetic provider, patients, and outcomes assessor were all blinded to patient allocation until analysis completion. 

All operations were performed by the same team of surgeons and one anesthesiologist. After standard monitoring including pulse oxymetry, noninvasive blood pressure, and electrocardiography, patients were premedicated with IV midazolam (0.02 mg/kg). Anesthesia was induced with IV propofol (1.5 mg/kg) and remifentanil infusion. Rocuronium (0.6 mg/kg) was given for orotracheal intubation. Patients were mechanically ventilated to maintain end-tidal CO_2_ between 35–40 mmHg at 50% inspired oxygen with air. Radial artery cannulation, central venous catheterization through the internal jugular vein, and urinary catheterization were performed. Anesthesia was maintained with an infusion of remifentanil (0.01–0.05 µg/kg/min) and desflurane within a range of bispectral index score 40–45, mean arterial pressure of 70–80 mmHg, central venous pressure of 8–12 cm H_2_O and urine output (UO) ≥ 1 mL/kg. Lactate Ringer’s solution or normal saline was infused at a rate of 10 mL/kg/h. When blood loss was more than 500 mL, 6% hydroxyethyl starch (Voluven^®^, Fresinius Kabi, Bad Homberg, Germany) was given. IV patient-controlled analgesia was started with fentanyl at a rate of 0.4 µg/kg/h for postoperative pain treatment.

Surgery began with anterior transperitoneal incision at the inferior border of 10th rib in the semi-lateral position. The kidney was dissected and mobilized within Gerota’s fascia, and was covered with an endopouch. 12g of IV mannitol was injected before arterial clamping. Renal vessels were then clamped with bulldog clamps in the hilum. An endopouch containing slushed ice was placed around the kidney for cooling. Before incising the mass, the kidney was kept covered with slushed ice for 5 min to obtain optimal renal core cooling. The tumor was resected, and suture renorrhaphy was performed. Then, renal vessels were declamped. Muscle, fascia, subcutaneous tissue, and skin were approximated, and no anticoagulant agent was given. 

Hemodynamics, operation and ischemia times, and fluid balance including transfusion were recorded. Tumor size was evaluated as the mean value of 3 dimensions of mass measured by computed tomography. The diuretic administration (≥ 1 time) was investigated for 3 days after surgery. The side effects associated with ketorolac or RIPC such as hypersensitivity, gastrointestinal troubles, bleeding tendency, uncontrolled hypertension, edema, palpitations, hypotension, liver dysfunction, or peripheral neuropathy were also evaluated.

Parameters for renal function were estimated; GFR (eGFR), serum creatinine (S_Cr_) and sodium (S_Na_), urinary creatinine (U_Cr_) and sodium (U_Na_), serum neutrophil gelatinase-associated lipocalin (NGAL), urinary N-acetyl-1-β-D-glucosoaminidase (NAG), and UO were all evaluated. Acute kidney injury (AKI) was assessed according to the AKIN criteria (S_Cr_ > 0.3 mg/dL or 150% increase or UO < 0.5 mL/kg within a 48 h) [12]. Blood or urine samples were collected at 7 time points; before induction (baseline), 10 min after anesthesia induction (postind), and 2, 12, 24, 48, and 72 h after renal artery declamping. Urinary NAG and serum NGAL were simultaneously collected at 3 time points; postind, 2 h, and 12 h after declamping. For urinary NAG, midstream of 3 mL of urine was stored at −70°C. For serum NGAL, 5 mL of blood was sampled from jugular venous line and centrifuged at 3000 rpm for 10 min. Then, the supernatant serum of 1.5 mL was gathered into an Eppendorf tube and frozen at −70°C for later analysis. UO was measured hourly with an indwelling catheter for 24 hours after surgery. After the urinary catheter was removed, hourly urine volume was estimated as a mean value of spontaneous voiding volume. Fractional excretion of urinary sodium (FE_Na_) was calculated using a formula of FE_Na_ (%) = (U_Na_ × S_Cr_)/(S_Na_ × U_Cr_) × 100. NAG was analyzed by colorimetric assay using a commercially available kit (N-Assay L NAG Nittobo, Nittobo Medical Co. Ltd., Tokyo, Japan). NGAL was measured by using a commercially available ELISA kit (SpectraMax 190, BIOPORTO, Sunnyvale, CA, USA).

For an eGFR, modified Cockcroft-Gault equation was used: GFR = [(140−age) × weight × 1.2]/S_Cr_ for men; GFR = [(140−age) × weight]/S_Cr_ for woman. The value was corrected for body surface area (BSA), then: eGFR = GFR × 1.73 m^2^/BSA. Lastly, the value was corrected using ischemia time and mass size on the assumption that these variables might influence S_Cr_. 

### Statistical Analysis

Statistical analysis was performed using PASW Statistics 20^TM^ (SPSS Inc., Chicago, IL, USA). Continuous data was analyzed with an unpaired t-test or Mann-Whitney U test. Categorical data was analyzed with a chi-square test. Repeat-measured data within the group were analyzed with a linear mixed model. When the interaction was statistically significant, a post-hoc test was performed and the *P* value was adjusted with Bonferroni correction. A *p* value of < 0.05 was considered statistically significant. Data are presented as mean ± standard deviation, median (range), or the number of patients.

## 3. Results

**Figure 1:**
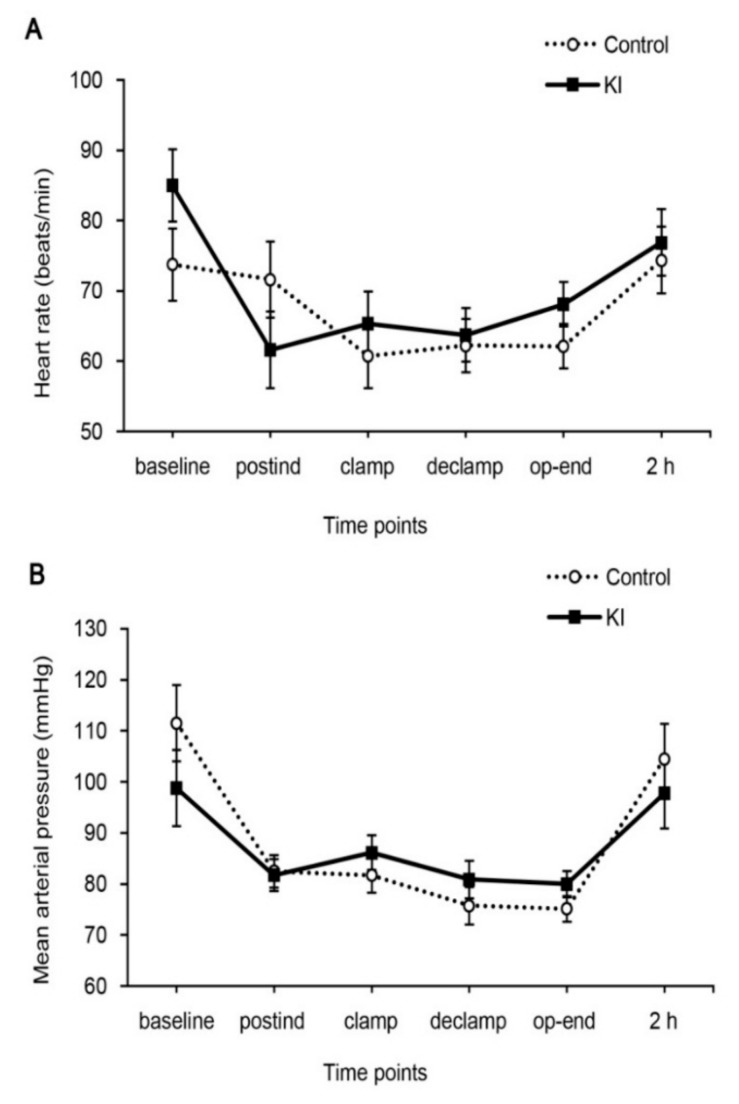
Intraoperative hemodynamics. (**A**) Heart rate and (**B**) mean arterial pressure. Baseline, before induction; postind, 10 min after anesthesia induction; clamp, 10 min before renal artery clamping; declamp, 10 min after renal artery declamping; op-end, end of surgery; 2 h, 2 h after renal artery declamping.

Patient characteristics and perioperative parameters were comparable between two groups (Table 1). The ischemia time was similar in two groups. Intraoperative heart rate and MAP were constantly maintained throughout the study period and there were no significant differences between the groups (Figure 1). Two patients from the control group were dropped from the study group because the bottles of serum samples were broken accidentally. 

### 3.1. Renal Function Profile

The number of patients administered a diuretic > 1 time, and duration of hospital stay, were similar between the two groups (Table 1). During the postoperative 48 h period, 1 patient from the KI group and 5 patients from the control group showed AKI defined as AKIN criteria (13% vs. 83%, *p* = 0.026) (Table 2). In the KI group, S_Cr_ was recovered to under baseline value at 72 h after declamping, while S_Cr_ in the control group was increased persistently until 72 h after declamping (Table 2). In all patients except 1 patient from the KI group, S_Cr_ was still higher than baseline value at 7 days after the operation. In 12 patients, S_Cr_ was still not recovered to baseline value 1 year after the operation. 

**Figure 2:**
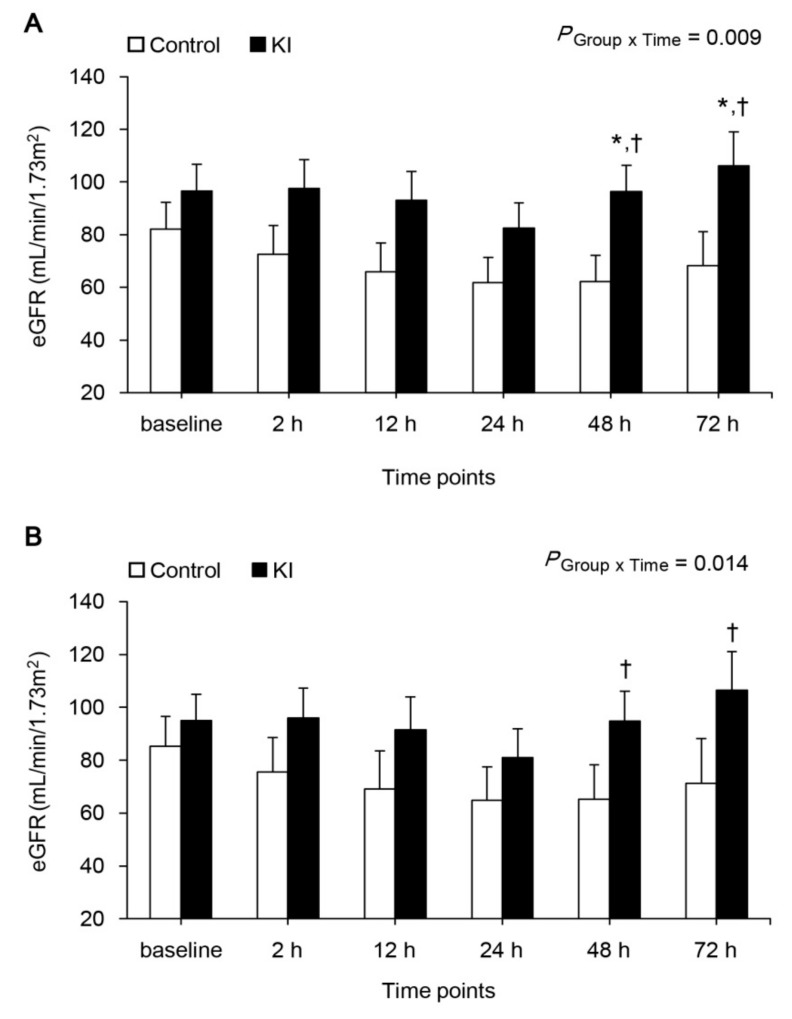
eGFR. (**A**) not corrected and (**B**) corrected by ischemia time and mass size. * *p* < 0.05 compared to control group, † *p* < 0.05 compared to 24 h; eGFR, estimated glomerular filtration rate; Baseline, before induction; 2, 12, 24, 48 and 72 h, 2, 12, 24, 48 and 72 h after renal artery declamping.

The eGFR is presented in Figure 2A. eGFR was persistently decreased until 24 h after declamping in both groups, but increased then significantly after 48 h after declamping in the KI group, while the control group still showed decreased eGFR until 72 h after declamping (*p* = 0.001 and *p* = 0.016 for 48 h and 72 h compared with 24 h, respectively). Additionally, eGFR was also significantly higher at 48 h and 72 h after declamping in the KI group compared with the control group (*p* = 0.025 and *p* = 0.044, respectively). The eGFR with corrected S_Cr_ using ischemia time and mass size is presented in figure 2B. The eGFR was also decreased to 24 h after declamping in both groups, but increased then significantly after 48 h hours after declamping in the KI group only (*p* = 0.002 and *p* = 0.027 for 48 h and 72 h compared with 24 h, respectively).

**Figure 3:**
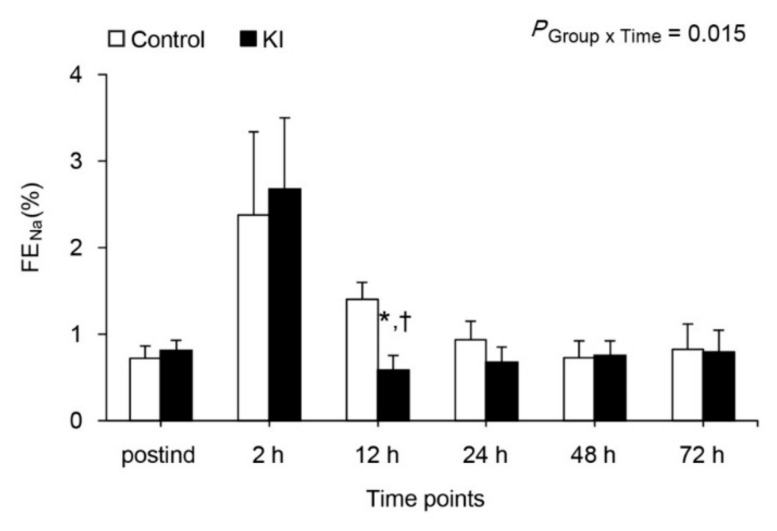
FE_Na_. * *p* < 0.05 compared to control group, † *p* < 0.05 compared to postind. FE_Na_, fractional excretion of sodium; postind, 10 min after anesthesia induction; 2, 12, 24, 48, and 72 h, 2, 12, 24, 48, and 72 h after renal artery declamping.

**Figure 4:**
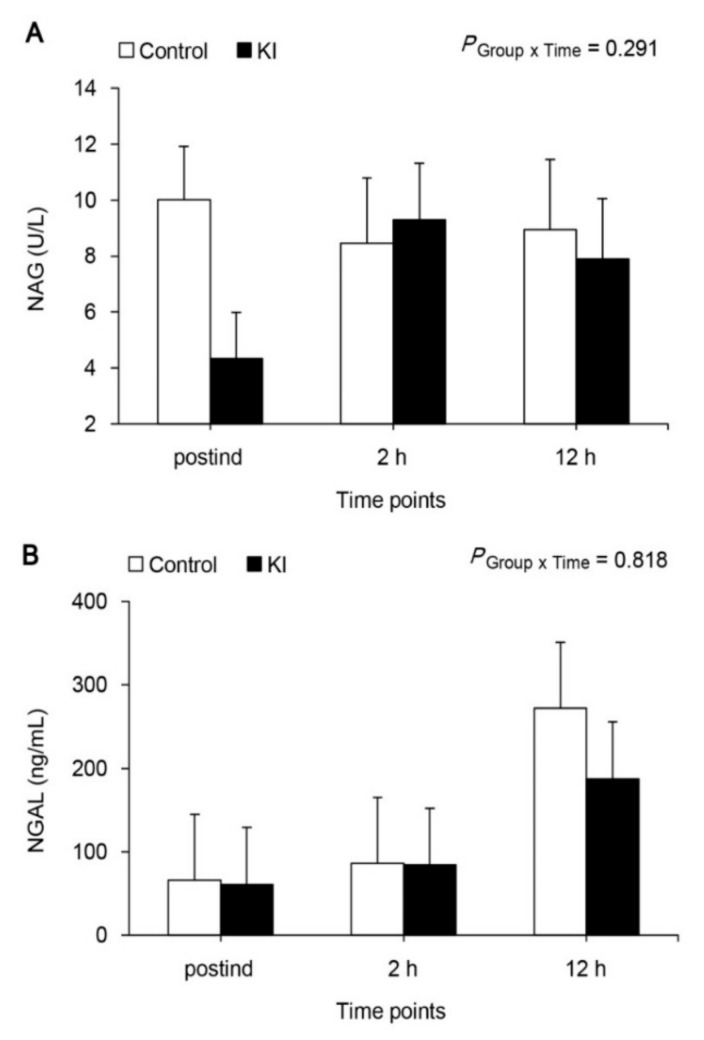
Urinary NAG (**A**) and serum NGAL (**B**) NAG, N-acetyl-1-β-D-glucosaminidase; NGAL, neutrophil gelatinase-associated lipocalin; postind, 10 min after anesthesia induction; 2 and 12 h, 2 and 12 h after renal artery declamping.

FE_Na_ was markedly increased at 2 h after declamping, and recovered to baseline value in both groups (Figure 3), but it was more significant at 12 h after declamping compared with postind in the KI group (*p* = 0.022). FE_Na_ was also significantly lower at 12 h after declamping in the KI group than in the control group (*p* = 0.009). The urinary NAG levels were within normal range at every measured time point, and were no different between the two groups (Figure 4A, *p* = 0.291). Serum NGAL levels were markedly increased at 2 h after declamping in both groups, but no difference was shown between the two groups (Figure 4B, *p* = 0.818). 

### 3.2. Complications

Postoperatively, one patient from the KI group and one patient from the control group showed diarrhea and fever. 

## 4. Discussion

The primary endpoint was to detect a difference of GFR in two groups after PN. Combined therapy of ketorolac administration and RIPC in the KI group prior to ischemia significantly attenuated persistent decreases of eGFR observed in the control group after ischemia-reperfusion-induced renal injury. It also significantly reduced incidence of AKI in the KI group compared with the control group according to AKIN criteria. Prompt recovery of FE_Na_ after a transient increase at 2 h after renal artery declamping was noticeable in the KI group, but not in the control group. Urinary NAG was similar between two groups and serum NGAL tended to increase in both groups after injury, however these did not reach a statistically significant difference between the two groups.

The renal cortex is quite susceptible to hypoxic injury and subsequent oxidative stress during an ischemia-reperfusion event [13]. In addition, inflammation contributes to the renal injury [7]. In acute ischemic injury, control of inflammation is helpful to progress to CKD, because the inflammation itself is associated with excessive production of extracellular matrix [14]. COX-2 has been found to be an immediate response gene that is highly inducible by inflammation, and thus many previous reports have examined the effect of the COX inhibitor on AKI associated with ischemia [8,15,16]. In line with this notion, we used ketorolac, a non-selective COX inhibitors to prevent AKI during PN. In conjunction with ketorolac, RIPC was also conducted during anesthesia induction during PN.

RIPC is a physiologic mechanism where distant tissues exposed to a brief period of nonlethal ischemia-reperfusion develop resistance to subsequent ischemic insult in critical organs such as the brain, spinal cord, heart, intestine, lung, liver and kidney [17,18]. Although protection mechanisms are complex and remain unclear, recent evidence indicates several theories; (1) humoral factors releasing, (2) neurogenic transmission involving in sympathomimetic stimulation, and (3) suppression of ROS and inflammatory mediators release [17].

Because inflammatory reaction appears to be initiated rapidly and evolves over the course of the injury, this early anti-inflammatory intervention may be more effective. Although our study has the limitation of an inability of dissecting out ketorolac effect from RIPC or vice versa, significant prevention of AKI in KI group (13% in KI group vs. 83% in control group) supports this hypothesis. 

After PN, renal function declined immediately after surgery (early), and reached its worst point (nadir), and then returned to a plateau (later) [19,20]. The median days to the nadir were at postoperative day 1 or 2 and the percentage of renal preservation to nadir was 77% [19,20]. In this study, eGFR in both groups decreased similarly immediately after surgery and reached nadir at 24 h after declamping, but exhibited different patterns since then. The KI group exhibited the improved renal function at 48 h and 72 h compared 24 h after declamping, and eventually showed recovery. However, the control group remained at nadir and did not recover.

FE_Na_ is regarded as the most accurate screening test to differentiate between prerenal and intrarenal disease origin in differential diagnosis of AKI. A low FE_Na_ (<1%) indicates decreased effective circulating volume (prerenal), which is transient and normalized within 7 days, whereas increased FE_Na_ (>1%) indicates acute tubular necrosis (intrarenal) and is persistent despite adequate treatment. In this study, ischemia by renal clamping seems to induce prerenal type AKI as shown by a low FE_Na_ (<1%), although diuretics, hydration, and antibiotics may interfere with this interpretation. 

NGAL is up-regulated very early in renal tubular cells after ischemic AKI, and is detectable within 2 h after insult, notably preceding the rise in S_Cr_. As an early predictive biomarker, serum NGAL has been used in various clinical settings [21]. In this study, serum NGALs in the KI group were lower compared to those in the control group, although not being significantly different, and eventually GFR improved in the KI group on postoperative day 2. Mori et al. [22] proposed a forest fire theory as the relationship between NGAL and S_Cr_. The rise in S_Cr_ is the mere passive result of a loss of functional nephrons, whereas an increase in NGAL is the consequence of a sustained production by “inflamed” tubular cells, in other words, a real-time indicator of ongoing damage. 

The COX-2 inhibitor and other NSAIDs are not always beneficial in preventing renal injury. In conditions with decreased effective circulating volume, the decrease of vasodilatory PG causes the constriction of renal arterial vessels, increasing the risk of acute renal insufficiency. However, in healthy hydrated individuals, renal PG does not play a major role in sodium and water homeostasis [23]. Renal blood flow and GFR are known to decrease significantly during the prolonged administration of COX-2 inhibitor [24]. Even if there is prolonged COX-2 inhibition, GFR decreases only when sodium intake is low because, in the setting of volume depletion, endogenous PG helps to maintain GFR possibly by dilating the afferent arteriole [25]. Indeed, in a clinical setting, ketorolac was not associated with the risk of renal failure when administered for early postoperative analgesia after PN or donor nephrectomy [26,27]. Ketorolac treatment ≤ 5 days did not increase the risk of renal failure [28]. In a large multicenter trial, ketorolac use after major surgery was safe even in patients with a history of renal insufficiency [29]. Collectively, it is unlikely that ketorolac treatment in our study is adversely associated with renal injury during PN.

While this study is ongoing, two research groups reported contradictory effects of RIPC on renal protection. Chen et al. used RIPC on one limb in both a donor and recipient during renal transplantation, but they did not find any evidence of renal protection [30]. By contrast, Jiwei et al. conducted RIPC on the lower limb during laparoscopic PN and showed short term renal protection, as measured indirectly via urinary retinol-binding protein, but not creatinine [10]. Therefore, our study is the first report stating that RIPC is beneficial in preventing AKI during PN based on solid evidence from measurements of S_Cr_, eGFR, FE_Na_, urinary NAG, and serum NGAL.

There were some limitations in this study. First, eGFR is a poor marker of the acute renal injury, in which creatinine is rapidly changing. Thus, eGFR may need to be followed over a longer period of time, considering that ATN from drugs might be a delayed process. Second, eGFR significantly changed, but urinary NAG or serum NGAL did not. This is partly because traditional biomarkers such as NAG and NGAL are not highly sensitive compared to novel biomarkers such as Kim-1 and vanin-1 [31,32], and urinary NGAL, not serum NGAL, may show any difference [33]. There needs to be an examination of the validity of novel biomarkers in this model. Third, the KI group can be regarded as having less potential for AKI in Table 1. In addition, there is a risk that improvement in eGFR is a result of a chance. These may be biased due to the small sample size. Further studies are needed to verify our findings in a larger sample size.

## 5. Conclusions

There is a possibility that combined therapy of ketorolac administration and RIPC prior to ischemic attack may alleviate renal dysfunction against ischemia-reperfusion injury and reduce the incidence of AKI in the patients undergoing open PN. Further study is required to clarify the beneficial effects of combined use of ketorolac and RIPC for postoperative renal function after PN.

## Figures and Tables

**Table 1:** Patient characteristics and perioperative parameters.

Variable	KI (*n* = 8)	Control (*n* = 8)	*p* Value
Age (years)	55 ± 12	61 ± 12	0.352
Weight (kg)	66 ± 8	70 ± 6	0.198
Height (cm)	165.2 ± 5.2	168.9 ± 6.2	0.216
BMI (kg/m^2^)	1.7 ± 0.1	1.8 ± 0.1	0.112
Sex (male)	6 (75%)	7 (88%)	>0.999
Medical history			
Hypertension	4 (50%)	6 (75%)	0.608
Atrial fibrillation	0	1 (13%)	>0.999
Old tuberculosis	0	1 (13%)	>0.999
Asthma	0	1 (13%)	>0.999
Operation time (min)	146 (99–217)	163 (70–197)	0.528
Anesthesia time (min)	190 (160–265)	201 (120–260)	0.875
Ischemia time (min)	29 (17–51)	30 (14–38)	0.462
Size of mass (cm)	3.5 (2.1–6.8)	2.8 (1.8–6.2)	0.461
Intraoperative fluid balance			
Crystalloid (mL)	1850 (1650–2600)	1800 (1000–2800)	0.874
Colloid (mL)	200 (0–1500)	500 (400–750)	0.451
Packed RBC (unit)	1 (13%)	0	>0.999
Urination (mL)	280 (48–422)	212 (100–315)	0.345
Bleeding (mL)	150 (60–1200)	350 (50–550)	0.206
Intraoperative medication			
Ephedrine	6 (75%)	6 (75%)	>0.999
Atropin	0	1 (13%)	>0.999
β-blocker	1 (13%)	0	>0.999
Betasin	1 (13%)	0	>0.999
Diuretic	2 (25%)	5 (63%)	0.315
Hospital stay (days)	4 (3–5)	6 (3–8)	0.118

Values are expressed as mean ± SD, median (range) or number (%); Diuretic was defined as the number of patients used diuretics ≥ 1 time during 3 postoperative days; BMI, body surface area; RBC, red blood cell.

**Table 2:** Acute kidney injury and serum creatinine.

Variable	KI (*n* = 8)	Control (*n* = 8)	*p* Value
Acute kidney injury *	1 (13%)	5 (83%)	0.026
Serum creatinine (mg/dL)			
Baseline	0.81 ± 0.18	0.96 ± 0.18	0.146
2 h	0.82 ± 0.22	1.06 ± 0.15	0.036
12 h	0.87 ± 0.25	1.18 ± 0.17	0.023
24 h	0.96 ± 0.25	1.29 ± 0.28	0.037
48 h	0.80 ± 0.21	1.28 ± 0.29	0.005
72 h	0.80 ± 0.18	1.14 ± 0.18	0.007

Values are expressed as mean ± SD or number (%); * Acute kidney injury was based on AKIN criteria; serum creatinine ≥ 0.3 mg/dL or 150% increase or urine output < 0.5 mL/kg/h× 6 h; Baseline, before induction; 2, 12, 24, 48 and 72, 2, 12, 24, 48 and 72 h after renal artery declamping.
